# No justice in a genocide: sexual and reproductive health and rights in Gaza

**DOI:** 10.1080/26410397.2025.2523095

**Published:** 2025-06-23

**Authors:** Cordelia Freeman, Hala Shoman

**Affiliations:** a Senior Lecturer, University of Exeter, Exeter, UK. *Correspondence*: c.freeman@exeter.ac.uk; b PhD Candidate, Newcastle University, Newcastle, UK

**Keywords:** conflict and crisis settings, Gaza, humanitarian crisis, Palestine, reproductive health, reproductive justice, sexual and reproductive health and rights

## Abstract

Since October 2023, Gaza, Palestine, has been the site of a humanitarian crisis due to violence from Israel, with numerous violations currently undermining sexual and reproductive health and rights. In this commentary, we detail these violations through the activist and theoretical framework of reproductive justice which centres on three tenets: the right to have children, the right not to have children, and the right to parent in safe environments and with dignity. Through examples such as the destruction of healthcare facilities, the lack of access to contraception or abortion and the total inability to parent safely, we document that all three tenets have been decimated through the systematic sexual, reproductive and gender-based violence inflicted on Palestinians by the Israeli security forces. We end the commentary with a discussion of “reproductive genocide” which we believe to be a term that accurately captures the wholesale decimation of life in Gaza.

## Résumé

Depuis octobre 2023, Gaza, en Palestine, est le théâtre d’une crise humanitaire due aux violences israéliennes, de nombreuses violations compromettant actuellement la santé et les droits sexuels et reproductifs. Dans ce commentaire, nous décrivons en détail ces violations à travers le cadre militant et théorique de la justice reproductive qui repose sur trois principes: le droit d’avoir des enfants, le droit de ne pas en avoir et le droit d’élever des enfants dans un environnement sûr et digne. À travers des exemples tels que la destruction des établissements de santé, le manque d’accès à la contraception ou à l’avortement et l’impossibilité totale d’élever des enfants en toute sécurité, nous démontrons que ces trois principes ont été anéantis par les violences sexuelles, reproductives et sexistes systématiquement infligées aux Palestiniens par les forces de sécurité israéliennes. Nous concluons ce commentaire par une discussion sur le « génocide reproductif » terme qui, selon nous, décrit avec justesse la destruction généralisée de la vie à Gaza.

## Resumen

Desde octubre de 2023, Gaza, Palestina, ha sido sede de una crisis humanitaria debido a la violencia de Israel, y numerosas violaciones actualmente socavan la salud y los derechos sexuales y reproductivos. En este comentario, explicamos en detalle estas violaciones por medio del marco activista y teórico de justicia reproductiva, que se centra en tres principios: el derecho a tener hijos, el derecho a no tener hijos y el derecho a criar los hijos en entornos seguros y con dignidad. Mediante ejemplos como la destrucción de establecimientos de salud, la falta de acceso a la anticoncepción o al aborto y la incapacidad total para criar a los hijos de manera segura, documentamos que los tres principios han sido diezmados por la violencia sistemática sexual, reproductiva y de género infligida al pueblo palestino por las Fuerzas de Defensa de Israel. Concluimos el comentario con un análisis del “genocidio reproductivo”, el cual consideramos ser un término que captura con precisión el diezmo total de la vida en Gaza.

Since October 2023, Gaza, Palestine, has been the site of a humanitarian crisis due to violence from Israel with numerous violations currently undermining sexual and reproductive health and rights (SRHR).^1^ In November 2024, the United Nations asserted that “Israel’s warfare in Gaza is consistent with the characteristics of genocide”^2^ and a March 2025 report by the UN details the systematic sexual, reproductive and gender-based violence inflicted on Palestinians by the Israeli security forces, including the deliberate destruction of healthcare facilities.^3^ Despite the supposed 2-month “ceasefire” in 2025, organisations such as the International Planned Parenthood Federation (IPPF), UNICEF and Médecins Sans Frontières have additionally documented the SRHR injustices faced by women, men and children.^4–6^ These include women undergoing caesareans without anaesthetic, a lack of care for adolescents including contraceptives, gender-based violence, and high rates of infant and maternal mortality. In this commentary, we as scholars of reproductive justice, one of whom is Palestinian, explore these violations of SRHR in Gaza through the lens of reproductive justice to uplift the research and documentation proving such violations and call on the SRHR community to recognise the destruction of reproductive justice in Palestine.

Reproductive justice is a scholarly and activist framework that was developed by black feminist activists and scholars in the United States as a critique of a prevailing individualised, “pro-choice” framing of reproduction.^7^ Reproductive justice centres on three core tenets: the right to have children, the right not to have children, and the right to parent in safe environments and with dignity. After a discussion of the long history of reproductive oppression in Palestine, this piece is structured around these three tenets to detail the denial of these rights during the genocide in Gaza. All three tenets are deeply intertwined and are central to how we can understand SRHR in practice. What we have seen in Gaza is the complete denial of bodily autonomy and that is a fundamental threat to the entire spectrum of sexual and reproductive health. Our goal with this commentary is to join other scholars in outlining the reproductive atrocities that have been taking place and continue to take place in Gaza and Palestine more broadly.^8–12^ Discussions of reproductive justice and of SRHR must include Palestine.

## Sexual and reproductive health and rights in Palestine

It is first imperative to note that the specific reproductive oppression seen since October 2023 in Gaza is not new, and that Israel has a decades-long history of attacking the SRHR of Palestinians. Particularly since the early 2000s, Israel has intensified its regime of closures and one impact of this has been the immobility of those attempting to travel for sexual and reproductive healthcare. Israel’s military occupation in Palestine creates obstacles to access medical facilities, forcing pregnant people to give birth at military checkpoints, with women and newborns dying in the process.^13^ This is just one factor that leads to the stark inequalities in sexual and reproductive health between Israel and Palestine. In 2017, the infant mortality rate was approximately six times higher in Palestine compared to Israel^14^ and the maternal mortality rate in the Gaza strip was 30/100,000 live births in 2014 compared to 7/100,000 live births in Israel.^15^ Gazans, particularly women and children, have experienced health-related vulnerabilities due to existing gender-based inequalities and discrimination and reinforced by the conflict.^16^

Beyond maternal and child health, the SRHR of adolescent girls and women have been impacted in numerous ways by decades of Israeli occupation. The 2005 UN Report of the Special Rapporteur on Violence against Women detailed how systematic Israeli violence has led to the detention, abuse, harassment, and humiliation of Palestinian adolescent girls and women.^17^ Gender-based and sexual violence and abuse are disproportionately experienced by women with disabilities, Bedouin women, internally displaced women and women and girls living in refugee camps.^18^ In addition, recurring shortages of contraceptives limit contraceptive choice, which negatively impacts fertility control.^19^ The more recent 2025 UN report shows that such crimes related to sexual, reproductive and gender-based violence have not only continued since 2023, but have been exacerbated.

These health disparities are underpinned by a clear history of socio-political discourses that dehumanise Palestinians and specifically target their reproductive potential. For instance, in 2008, after a military attack by Israel in the Gaza Strip, Israeli soldiers produced t-shirts with an image of a pregnant Palestinian woman centred in the crosshairs of a rifle, captioned with “1 shot 2 kills”.^20^ Palestinians are viewed as a “demographic threat” by Israel as a state and the killing of women and children is a strategy to prevent the existence of future “terrorist” Palestinians to uphold Israel's settler-colonialist claims to land and identity.^21^ As one Israeli lawmaker, Ayelet Shaked, from the far-right Jewish Home party stated in 2014, in a subsequently deleted Facebook post, “Behind every terrorist stand dozens of men and women, without whom he could not engage in terrorism. They are all enemy combatants, and their blood shall be on all their heads. Now this also includes the mothers of the martyrs, who send them to hell with flowers and kisses. They should follow their sons, nothing would be more just. They should go, as should the physical homes in which they raised the snakes. Otherwise, more little snakes will be raised there.”^21^^22^

As these examples illustrate, the situation of SRHR and of reproductive justice was already dire in Palestine before October 2023, and this has since been further exacerbated through extreme attacks. We next explore these strategies of reproductive oppression in Gaza since October 2023 through the three key tenets of reproductive justice: the right to have a child, the right to not have a child, and the right to parent a child or children in safe and healthy environments.

## The right to have a child in Gaza

First, the right of people to have a child has been almost entirely decimated in Gaza since October 2023. Genocide not only seeks to eradicate a group of people but also erases their future by destroying their ability to reproduce and have children. This includes the direct killing of individuals and the loss of partners with whom survivors might have had children. The violence thus extends beyond immediate present death, severing the potential for reproduction and continuity. Beyond these direct violations, people in Gaza have also had the right to have children taken away from them through the obstacles created by the genocide that mean they cannot become pregnant or sustain a pregnancy.

Clear evidence shows high rates of miscarriage in Gaza due to malnourishment, dehydration and high stress that are all consistent with famine conditions. This is all evident in the reported 300% increase in rates of miscarriage since October 2023.^12^ Anaemia is very high in Gaza due to food scarcity, increasing the risks of complications in pregnancy, and increasing the likelihood of premature births, low-birth weights, and developmental delays.^23^ Without essential medical care, water and food, pregnant people in Gaza are facing severe risks to being able to carry a pregnancy to term. Even where they are able to carry to term, exposure to white phosphorus and high metal loads in environmental contaminants introduced by weaponry is increasing serious birth defects which will mean newborns are unable to survive.^23,24^ Being exposed to armed conflict during pregnancy leads to higher rates of miscarriage, stillbirths, prematurity, congenital abnormalities, and other negative health outcomes.^9^ This is all exacerbated by the reality that obstetrics and gynaecology specialists have been killed, detained and displaced, resulting in a lack of support and equipment for pregnant Gazans.^25^

Moreover, for those desiring to become pregnant in Gaza, in addition to the above conditions that impede fertility, there is a total lack of assisted reproductive technologies. The landscape of assisted reproductive technologies was already complex for Palestinians with reproductive border-crossings common for Palestinians to access fertility clinics in Israel.^26^ The possibility of receiving fertility care is now non-existent as in late 2023, the Al Basma in-vitro fertilisation clinic was shelled, thereby destroying the 4000 embryos stored there and leaving communities with no access to fertility services.^25^

However, much of this evidence centres on the capacity for women to become and remain pregnant during a genocide. It is imperative that Gazan men are included in analyses of SRHR and their right to have a child is also being denied through the genocide. The differences between the rights of Palestinian and Israeli men to father children could not be more stark. In July 2024, the BBC reported that “Since 7 October, sperm has been retrieved from nearly 170 young men – both civilians and soldiers – according to the Israeli health ministry”.^27^, np.^28^ This posthumous sperm retrieval programme gives a greater right to dead Israeli men to become fathers than to Gazan men.

## The right to not have a child in Gaza

Second, the right not to have a child is simultaneously being eradicated in Gaza. This is an exacerbation of ongoing issues related to humanitarian contexts whereby discriminatory gender norms and expectations of early marriage to produce children have particularly impacted adolescent girls.^29^ However, in recent months it has become almost impossible to access contraceptives.^30^ Contraceptive pills were being widely used to delay menstruation in a context where people could not access menstrual products or private bathrooms and the depletion of stocks means people will experience increased bleeding and also the increased potential for unwanted pregnancies.^25^ Other contraceptive methods such as intrauterine devices and condoms are also unavailable because distribution centres and reproductive health clinics have been destroyed, with impacts for unwanted pregnancy as well as sexual health.^25^ Emergency contraception, or the morning-after pill, is also inaccessible in Gaza, meaning that those who have consensual sex or are raped in these conditions have no recourse to reduce their chances of experiencing an unwanted pregnancy.^31^

For those who do experience an unwanted pregnancy in Gaza, there is no access to abortion. The number of unwanted pregnancies in Gaza is increasing given the risks of pregnancy, childbirth, and the prospect of raising a child amidst the current violence. Pregnancies that may have been much desired before October 2023 now feel unfathomably dangerous. There are currently no pathways to accessing a safe abortion and reports show people resorting to unsafe methods, thereby increasing risks of maternal mortality.^25,30^ Previous to October 2023, abortion was illegal in Palestine unless the pregnant person’s life was at risk, and there were no government services offering support for the termination of pregnancies.^32^ However, there were United Nations and governmental clinics in the Gaza Strip that offered sexual and reproductive services and abortions were accessed in practice through private clinics, Israeli clinics, or through self-managed abortions.^32,33^ Self-managed abortions with pills are highly safe with access to accurate information,^34^ and this should be available in the region, especially given that clinics are now inaccessible.

Finally, the rape of Palestinians by Israelis has the potential to lead to unwanted pregnancies. In February 2024, the United Nations High Commissioner for Human Rights (UHCHR) communicated two Palestinian women's reports of being raped in Israeli detention.^35^ The true scale of these rapes and the potential they have to result in pregnancies is unknown. Without access to contraception, abortion, or freedom from rape, there can be no right to not have a child in Gaza.

## The right to parent a child or children in safe and healthy environments in Gaza

Third, reproductive justice is “the struggle for the collective conditions for sustaining life and persisting over time amid life-negating structural forces, and not just the right to have or not have children” and these conditions are no longer existent in Gaza.^36^ Right from the start of life, the 183 women who were giving birth every day in late 2023 were forced to bring children into severely dangerous conditions.^9^ Complications are most likely within the first 24 hours of birth, and due to the scarce and overburdened or non-existent maternal health facilities, mothers are being discharged almost immediately and are unable to access postpartum care.^30^ Even when healthcare services are available, not all people have the fuel to access them.^5^ Therefore, it is now completely impossible to give birth in safety and with dignity in Gaza where women are left “to deliver babies outside, in unsanitary conditions, without the assistance of midwives or doctors”.^10^

Continuing into infancy, these same problems obliterate the right to parent children safely. Previous to the current genocide, vaccinations for infants were available widely and free of charge and these are now inaccessible. Due to blockades and the targeting of hospitals, medical supplies such as maternity kits and anaesthetics are scarce and this is not accidental.^10^ Targeted violence and the denial of access to services and supplies is a deliberate strategy by Israel. Lack of maternal health services means parents cannot access health checks for infants and urgent care cannot be provided, as shown by the 130 premature babies who were being kept alive in incubators in November 2023 that were at constant risk of direct attack or losing their vital electricity supply.^37^ The IPPF^4^ has additionally reported cases of women unable to breastfeed their babies due to their malnutrition and anxiety.

If these babies continue into childhood, they are in grave danger of being murdered with UN Secretary General Antonio Gutierrez calling Gaza a “graveyard for children”^38^ and UNICEF Director Catherine Russell calling it “the most dangerous place in the world to be a child”.^6^ As a December 2024 report by Airwars^39^ showed, families are being killed together in their homes on an unprecedented scale with 90% of women and children killed being murdered in homes and 95% of women being killed dying alongside at least one child. Even beyond direct murder, there is mass exposure to life-threatening toxicity from the use of chemical weapons and there are indications that Israeli forces have been using white phosphorus in their attacks in Gaza, which is highly concerning given its increased harm on children.^8^ Moreover, the mass destruction of homes, schools, agriculture, places of worship, and many other crucial facets of life and society all eradicate the right to parent a child or children in safe and healthy environments. As *The Guardian* reported in December 2024, one study showed that 96% of children in Gaza feel that their death is imminent and nearly half wish to die.^40^

## Reproductive injustice or reproductive genocide?

The examples illustrating this commentary highlight the reproductive injustices that have a long history in Palestine but have been exacerbated in Gaza since October 2023. This exacerbation is to the extent that all three tenets of reproductive justice have been entirely obliterated. Therefore, in these circumstances, is it even possible to speak of reproductive justice? For these reasons, the Palestinian Feminist Collective^41^ uses the term “reproductive genocide” in an attempt to capture the wholesale decimation of life in Gaza. Safety from mass murder and genocide is a prerequisite to reproductive justice and reproductive justice is impossible without including Palestinian liberation.

As powerfully stated in a letter signed by 411 repro-scholars, workers, and activists, called “Resistance is Fertile”^42^ “[r]eproductive justice means allowing all Palestinians, from the river to the sea, to build and rebuild the infrastructures of life and social reproduction they need to live a life worth living, free from bombs and colonial dispossession. There can be no reproductive justice when it does not count for everyone, Jewish and Palestinian people alike. There can be no reproductive justice without a free Palestine”. While this commentary draws on the initial sources emerging from the catastrophe, further detailed research is needed to document and understand the reproductive genocide affecting men, women, adolescents and children with an intersectional analysis.^43^ The SRHR community and feminists more broadly must recognise the destruction of reproductive justice in Palestine by the Israeli regime and join the collective struggle for freedom and justice in Palestine.^44,45^

## Author contributions

Conceptualisation: CF, HS. Writing – original draft: CF. Writing – review & editing: CF and HS.

## Provenance statement

This article was not commissioned and has been through external peer review.
